# Increased expression of transient receptor potential channels and neurogenic factors associates with cough severity in a guinea pig model

**DOI:** 10.1186/s12890-021-01556-w

**Published:** 2021-06-02

**Authors:** Mengyue Guan, Sun Ying, Yuguang Wang

**Affiliations:** 1 Department of Respiratory Medicine, Beijing Hospital of Traditional Chinese Medicine, Capital Medical University, No. 23rd Art Museum Backstreet, Dongcheng District, Beijing, 10010 China; 2 Department of Immunology, School of Basic Medical Sciences, Capital Medical University, No. 10th Xitoutiao, You’anmenwai Street, Fengtai District, Beijing, China

**Keywords:** IPF-related high cough sensitivity, Jugular ganglion, Nodal ganglion TRP, TRPA1, TRPV1

## Abstract

**Background:**

Previous studies suggest that transient receptor potential (TRP) channels and neurogenic inflammation may be involved in idiopathic pulmonary fibrosis (IPF)-related high cough sensitivity, although the details of mechanism are largely unknown. Here, we aimed to further explore the potential mechanism involved in IPF-related high cough sensitivity to capsaicin challenge in a guinea pig model of pulmonary fibrosis induced by bleomycin.

**Methods:**

Western blotting and real-time quantitative polymerase chain reaction (RT-qPCR) were employed to measure the expression of TRP channel subfamily A, member 1 (TRPA1) and TRP vanilloid 1 (TRPV1), which may be involved in the cough reflex pathway. Immunohistochemical analysis and RT-qPCR were used to detect the expression of neuropeptides substance P (SP), Neurokinin-1 receptor (NK1R), and calcitonin gene-related peptide (CGRP) in lung tissues. Concentrations of nerve growth factor (NGF), SP, neurokinin A (NKA), neurokinin B (NKB), and brain-derived neurotrophic factor (BDNF) in lung tissue homogenates were measured by ELISA.

**Results:**

Cough sensitivity to capsaicin was significantly higher in the model group than that of the sham group. RT-qPCR and immunohistochemical analysis showed that the expression of TRPA1 and TRPV1 in the jugular ganglion and nodal ganglion, and SP, NK1R, and CGRP in lung tissue was significantly higher in the model group than the control group. In addition, expression of TRP and neurogenic factors was positively correlated with cough sensitivity of the experimental animals.

**Conclusion:**

Up-regulated expression of TRPA1 and TRPV1 in the cough reflex pathway and neurogenic inflammation might contribute to the IPF-related high cough sensitivity in guinea pig model.

**Supplementary Information:**

The online version contains supplementary material available at 10.1186/s12890-021-01556-w.

## Background

Idiopathic pulmonary fibrosis (IPF) is a chronic progressive fibrotic lung disease, the etiology of which is still unknown [1]. The main clinical manifestations include cough, progressive dyspnea, and fatigue, while the survival period after diagnosis is approximately 2–5 years [2, 3]. Normally, cough, particularly chronic irritant dry cough, is a common clinical symptom of IPF that occurs in approximately 73–86% of IPF patients [4]. The symptoms are severe during the day and seriously affect the quality of life of affected patients [5]. In addition, cough in patients with IPF is an independent predictor of the prognosis of the disease [4], often refractory, while traditional cough medicines have poor efficacy [6].

The pathophysiological mechanism underlying IPF-related cough remains unclear, although some studies suggest that it might relate to neurogenic inflammation, transient receptor potential (TRP) channels, and increased cough sensitivity [7]. Neurogenic inflammation refers to the activation of sensory neurons when they are thermally or chemically stimulated, which leads to the release of substance P (SP), calcitonin gene-related peptide (CGRP), neurokinin A (NKA), and other inflammatory mediators, and consequently to inflammatory reactions such as local tissue edema [8]. It has been shown that the main mediators of neurogenic inflammation such as CGRP and SP play important regulatory roles in the cough reflex [9]. When neurogenic inflammation occurs, neurons release bradykinin, nerve growth factor (NGF), and other inflammatory mediators, which can not only directly stimulate and activate cough receptors in the airway mucosa, but also increase cough sensitivity to normal subliminal stimuli [10].

As cough receptors, TRP channels of the respiratory system, especially TRP channel vanillin subfamily member 1 (TRPV1) and TRP channel subfamily A member 1 (TRPA1), are sensitive to thermal and chemical stimulation, which contribute to the pathogenesis of chronic cough [11]. It has been shown that up-regulated expression of TRPA1 and TRPV1 can increase cough sensitivity, which can be indirectly sensitized by a series of inflammatory mediators such as bradykinin and NGF [12]. Furthermore, when the cough sensitivity of children with chronic cough increases, levels of CGRP in bronchoalveolar lavage fluid (BALF) also increase [13]. Patients with IPF also have increased cough sensitivity to chemical stimuli (inhalation of capsaicin or SP) and increased levels of neurotrophins in sputum [14]. We have previously shown that expression of TRPA1 and TRPV1 in the lungs of a guinea pig model of bleomycin-induced pulmonary fibrosis was elevated, which was companied with increased cough sensitivity [15]. This suggests that TRP channels and neurogenic inflammation might be involved in the pathogenesis of IPF-associated high cough sensitivity.

It is known that every involuntary cough is composed of a complete reflex arc [16]. After the sensory nerve endings are stimulated, the nerve impulse travels along the vagus nerve to the brainstem cough center where the signals are integrated and then transmitted to the effectors (e.g., diaphragm, larynx, chest, abdominal muscles) via the efferent nerve, causing cough [17]. Airway afferent nerves related to cough reflex mainly originate from the nodose ganglia and jugular ganglia, both of which express cough receptors such as TRPA1 and TRPV1 [18]. However, it is still not clear whether there are changes in the expression of TRP channels in the jugular ganglia and nodose ganglia in IPF-related cough, or in the expression of the neurogenic indicators SP, Neurokinin-1 receptor (NK1R), and CGRP in lung tissues.

To further address these unknown issues, we aimed to explore the expression of TRPA1 and TRPV1 in the jugular ganglia and nodose ganglia and that of neurogenic indicators such as SP, CGRP, and NK1R in the lungs using an established guinea pig cough model of pulmonary fibrosis induced with bleomycin. We hypothesized that the increased cough sensitivity in the bleomycin-induced pulmonary fibrosis guinea pig model is associated with the upregulation of TRPA1 and TRPV1 in the jugular ganglia and nodose ganglia in the cough afferent pathway, which is related to the occurrence of neurogenic inflammation in the lungs.

## Methods

### Reagents and materials

Primary rabbit antibodies against TRPA1 (NB110-40763), CGRP (NBP2-88980), NK1R (NB300-119), TRPV1 (OST00029W), and SP (Ab14148) were purchased from Novus Bio (Littleton, MA), Invitrogen (Carlsbad, CA), and Abcam (Cambridge, MA), respectively.

A commercial hydroxyproline assay kit was purchased from the Jiancheng Bioengineering Institute (Jiangsu, China), while bleomycin and capsaicin were purchased from Hisun-Pfizer Pharmaceuticals (Zhejiang, China) and Sigma-Aldrich (Seattle, WA), respectively.

To measure concentrations of NKA (MC14120), neurokinin B (NKB, MC14120), matrix metallopeptidase-9 (MMP-9, MC14143), brain-derived neurotrophic factor (BDNF, MC14115), tissue inhibitor of metalloproteinase-1 (TIMP, MC14142), NGF (ML028147-JK48) and fibroblast growth factor 2 (FGF2, ML028139-GC96T) in lung tissue homogenates of guinea pig models, ELISA kits were purchased from the Meichenlianchuang Bioengineering Institute (Beijing, China) and Boaokaimei Bioengineering Institute (Beijing, China).

For measuring mRNA expression, PureLinkTM RNA Mini and One-Step PrimeScript TM real-time polymerase chain reaction (RT-qPCR) kits were purchased from Thermo Fisher Scientific (Carlsbad, CA) and TaKaRa (Dalian, China), respectively, while the encoding primers were synthesized by Sangon (Shanghai, China) (Table 1).

**Table 1:** Sequences of the genus-specific primers

GAPDH	Forward: 5′-CAACTACATGGTCTACATGTTC -3′
	Reverse: 5′-CTCGCTCCTGGAAGATG -3′
TRPV1	Forward: 5′-CAGTGGGAAGATTGGGGTCT-3′
	Reverse: 5′-AAGTCCTCGGCCACATTGTA-3′
TRPA1	Forward: 5′-GCCAGGAACAGCTTTCAACT-3′
	Reverse: 5′-ACAAACAAGGGCAGCACAAA-3′
SP	Forward: 5′-GGGTTATGAAAGGAGTGCCA-3′
	Reverse: 5′-CTCTGAGGGAGGAGTCAGAA-3′
NK1R	Forward: 5′-ACTGGAGCTCTGAGAAGTGT-3′
	Reverse: 5′-CTTAGGTCACACAGCATGGG-3′
CGRP	Forward: 5′-CTCTACTCCAAGACCTCGCC-3′
	Reverse: 5′-CACTGGCCTTCATCTGCATG-3′

The total collagen content in the lung tissues of the experimental animals was measured using hydroxyproline assay purchased from Jiancheng Bioengineering Institute (Jiangsu, China), according to the manufacturer’s instructions.

### Animal models and experimental design

All animal experimental procedures in the present study were performed in accordance with the Guide for Care and Use of Laboratory Animals (8th edition, released by the National Research Council, USA) and approved by the Animal Experiment Committee of Laboratory Animal Center, Academy of Military Medical Sciences (approval number: IACUC-DWZX-2020-048).

Pathogen-free, male Hartley guinea pigs, weighing 250–300 g, were purchased from Beijing Weitong Lihua Experimental Animal Technology Company (Beijing, China) and maintained under specific pathogen-free conditions. After 5 days of adaptive feeding, guinea pigs with abnormal cough sensitivity were excluded, while the rest of the animals were randomly assigned to the sham operation and model groups (~ 16–18 per group) using the random number table method and subjected to the cough sensitivity test. Each group of guinea pigs served as an experimental unit.

A guinea pig laryngoscope (HRH-HAG5; Beijing Hui-Rong-He Technology Company) (Additional file 1: Fig. 1A-a) and a pulmonary micro-liquid nebulizer (HRH-MAG4; Beijing Hui-Rong-He technology company) (Additional file 1: Fig. 1A-b) were used to examine the throat, as these allow the operators to clearly visualize the structure of the larynx and respiratory tract, as well as to directly and precisely deliver the atomized drug into the lungs of guinea pigs during inhalation. In brief, guinea pigs were subjected to fasting for 8 h and anesthetized using intraperitoneal injections of 1% pentobarbital sodium solution (Sigma, P-010, St. Louis, MO, USA) at a dose of 30 mg/kg. The guinea pigs were then placed on the surgery table (HRH-HAG7; Beijing Hui-Rong-He Technology Company, Beijing, China) in a supine position with the upper teeth and limbs secured (Additional file 1: Fig. 1Ac). Additionally, the angle between the operating table and tabletop was adjusted to 45°, that is, the mouth, throat, and body of the animal, were placed in a straight line to allow a convenient and quick delivery of aerosol drugs into the trachea. Approximately 0.2 mL of PBS (sham operation group) or bleomycin (11.428 mg/mL; model group) was evenly distributed throughout the lungs of each animal (calculated on the basis of the body weight, 0.7 μL/g). Following the administration of the bleomycin or PBS, guinea pigs were placed on electric blankets to keep them warm. Animals were returned to the rearing room once they were awake and able to move normally.

### Cough induction

**Fig. 1:**
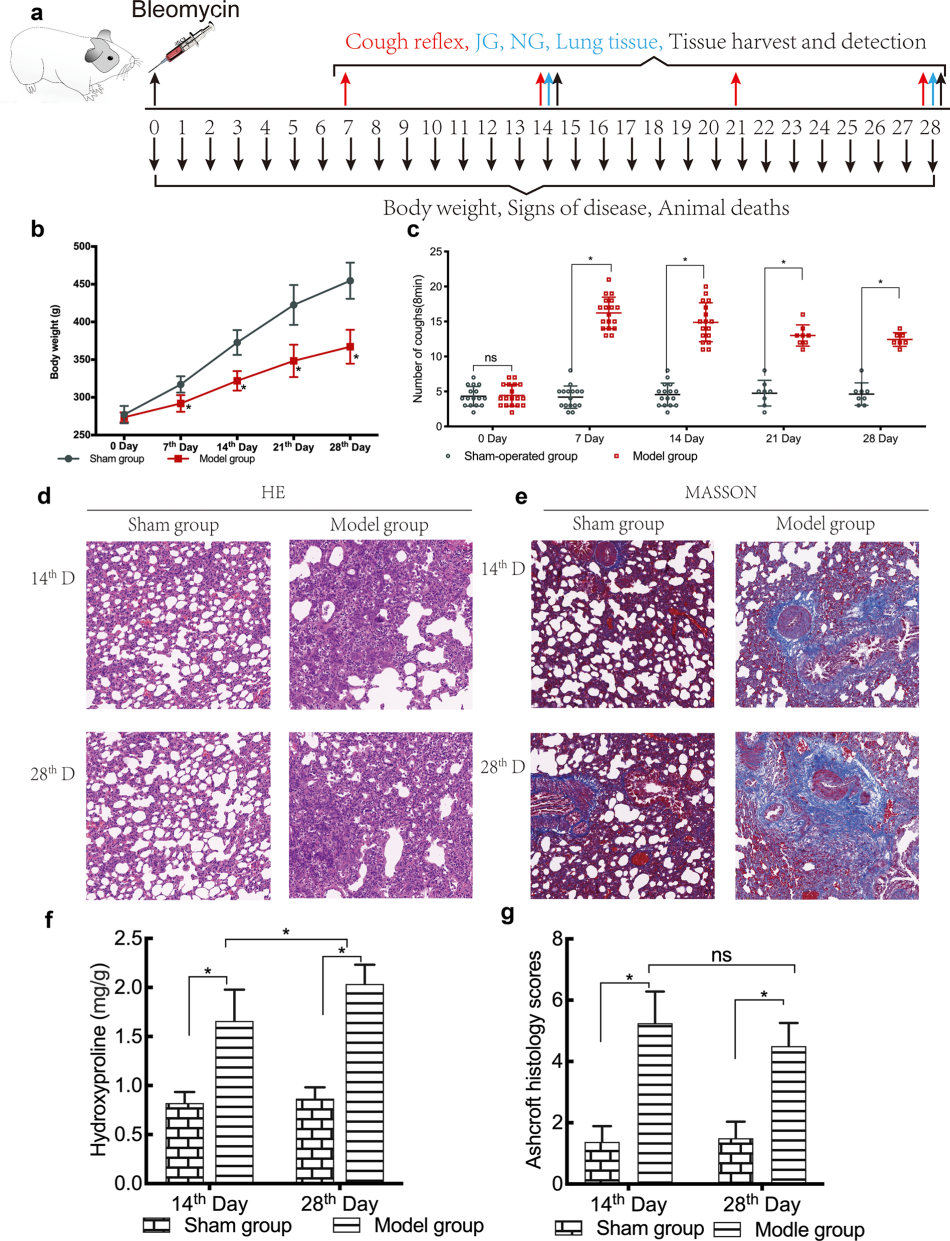
Body weight changes, pulmonary inflammation, fibrosis, and cough reflex sensitivity in guinea pigs. The cough reflex index was evaluated on the 7th, 14th, 21st, and 28th day after intratracheal administration of bleomycin (n = 16–18 animals/each time point on days 7 and 14; n = 7–8 animals/each time point on days 21 and 28). Samples were collected on the 14th and 28th das after the cough reflex test (**a**). Body weights of guinea pigs were measured before and after the administration of bleomycin (**b**). Cough responses to 0.75 μM capsaicin were recorded prior to and on the 7th, 14th, 21st, and 28th day after bleomycin administration (**c**). On the 14th and 28th day after the intratracheal administration of bleomycin, the lung tissues were collected after animals were sacrificed. The lung tissue sections were stained by H&E (**d**) and Masson’s stain (**e**) to evaluate inflammation and fibrosis. Additionally, the concentration of collagen in lung tissues was measured (**f**). The results of Masson staining were quantified using the Ashcroft scale (n = 6/group/per time point) (**g**). The data are expressed as the mean ± SD. *P < 0.05

On the 7th, 14th, 21st, and 28th days after the administration of bleomycin, the cough reflex sensitivity of each guinea pig (Fig. 1a) was evaluated in a conscious and unconstrained state as described previously [19–21]. Briefly, the animals were placed in a closed cylindrical container prepared in our laboratory, into which room air was continuously filled using a micropump nebulizer (OMRON, Japan). Capsaicin solution (75 μM; Sigma, St. Louis, MOA) was delivered to the container via an aerosol pump using a micropump nebulizer plastic tubing [15]. The guinea pigs were exposed to capsaicin in the container for 3 min and observed for a further 5 min [19].

The number of coughs during the first 3 min and the subsequent 5 min was counted by two trained observers. Coughs were counted by acoustical monitoring of the sounds made by the animals and continuous visual monitoring of the characteristic animal postures, including mouth-opening and abdominal movements associated with coughing. All animals were monitored daily to determine the changes in weight and to observe the clinical symptoms. The number of deaths was recorded.

### Sample preparation

Guinea pigs were randomly selected from the sham operation and model groups (n = 7–8/time point) on the 14th and 28th days after bleomycin administration. Following the induction of anesthesia with an intraperitoneal injection of 1% pentobarbital sodium solution at a dose of 30 mg/kg, the guinea pigs were sacrificed using the femoral artery bleeding method. The right lungs were isolated, placed into cryotubes, quickly frozen in liquid nitrogen, and transferred to a − 80 °C refrigerator until further analysis by western blotting, ELISA, and RT-qPCR. The left lungs of the animals were fixed in 4% paraformaldehyde for > 24 h and embedded in paraffin after dehydration for subsequent staining with hematoxylin and eosin (H&E), Masson, and immunohistochemistry.

### Histopathological analyses

Paraffin blocks were sliced into section of 5 μm thickness for histological analysis. Sections were stained using H&E and Masson’s stain to evaluate the degree of inflammation and the presence of fibrosis, respectively. The Ashcroft scale was used to measure the degree of bleomycin-induced lung fibrosis, as reported by Ashcroft et al. and Hübner et al. [22, 23]; six stained fields were randomly selected from the lung tissue sections obtained from each guinea pig and analyzed using the Ashcroft scale.

### Immunohistochemistry

Paraffin sections were deparaffinized in xylene and rehydrated in a series of graded alcohols, while endogenous peroxidase was quenched with 3% hydrogen peroxide (H_2_O_2_) for 10 min. For antigen retrieval, sections were placed in citrate buffer (pH = 6), microwaved (Sharp, R-331ZX) for 3 min at 95 °C, and allowed to cool down for 20 min at 26 °C; this process was repeated once. After blocking with a blocking reagent for 30 min at room temperature, sections were incubated with primary antibodies against SP (1:5000), NK1R (1:2500), and CGRP, (1:3000) overnight at 4 °C. After washing, sections were then incubated with poly-horseradish peroxidase (HRP) anti-rabbit IgG secondary antibodies (1:1000) or poly HRP anti-mouse IgG secondary antibodies (1:1000) for 20 min. Immunoreactivity was developed with DAB (3,3′-Diaminobenzidine tetrahydrochloride, a substrate of HRP) for 20 min. After washing, sections were counterstained with hematoxylin.

Immunoreactivity for SP, NK1R, and CGRP was visualized and quantified using an Olympus DP70 microscope (Olympus Corporation, Tokyo, Japan) and a NanozoomerS210 whole slide scanner (Hamamatsu Photonics, Hamamatsu City, Japan). A total of six visual fields in sections per animal were chosen randomly and images were analyzed using Image-Pro Plus 6.0 software.

### Western blotting

Western blotting was performed to analyze expression of proteins in the jugular ganglion and nodular ganglion of the animals. Ganglion tissues were homogenized in radioimmunoprecipitation assay (RIPA) lysis buffer supplemented with a protease inhibitor cocktail (Sigma-Aldrich) on ice. After centrifugation of the samples at 12,000 rpm (ThermoFisher, legend micro 21r) for 10 min, supernatants were collected.

Protein samples were denatured by boiling in sodium dodecyl sulfate (SDS) loading buffer and separated by SDS–polyacrylamide gel electrophoresis on a 10% polyacrylamide gel. Proteins were subsequently transferred onto nitrocellulose membranes (Bio-Rad, Hercules, CA), which were treated with rapid block buffer (Sangon, Shanghai, China, C510053-0020) for 10 min to block nonspecific binding. Subsequently, membranes were incubated overnight with primary antibodies against TRPV1 and TRPA1 at 4 °C. After washing three times (5 min for each), blots were incubated with a HRP-conjugated secondary antibody at a dilution of 1:2,000 for 60 min at 37 °C. Specific proteins were detected using an enhanced chemiluminescence reagent (Amersham Biosciences, Piscataway, NJ). The intensities of the individual bands were quantified using ImageJ software. All assays were performed independently and in triplicate.

### ELISA

ELISA was performed for measuring concentrations of NKA, NKB, BDNF, and NGF in lung tissue homogenates of animals according to the manufacturer’s instructions.

### Real-time quantitative PCR

Total RNA samples were isolated from the jugular ganglion, nodular ganglion, and lung tissues using TRIzol reagent (Invitrogen, Carlsbad, CA), according to the manufacturer’s instructions. The QuantiFast SYBR Green RT-PCR kit (QIAGEN, Düsseldorf, Germany) was used for PCR amplification, while the mRNA expression levels of TRPV1, TRPA1, SP, NK1R, and CGRP were quantified by RT-qPCR using different primers (Table 1). Each sample was assayed in triplicate.

### Statistical analyses

All statistical analyses were performed using SPSS version 26.0 (SPSS Software, Armonk, NY). Data are expressed as mean ± standard error of the mean (SEM). The time-course results were compared among multiple groups using two-way analysis of variance (ANOVA), followed by the Sidak test. The other results were compared between the groups using one-way ANOVA, followed by Dunnett’s multiple comparison test or two-tailed unpaired t-tests. Correlations of the cough numbers and relevant indexes measured were analyzed using a Pearson correlation coefficient. *P*-values < 0.05 were considered to be statistically significant.

## Results

### Animal deaths, signs of disease, and changes in body weight

There were no deaths in the sham group; however, three deaths occurred in the model group, on the 13th, 16th, and 21st day after the administration of bleomycin.

Guinea pigs in the sham operation group had flexible activities, bright and clean hair, a normal dietary intake, and a steady increase in body weight throughout the period of experiment. In contrast, those in the model group suffered from rapid breathing, reduced food and water intake, less movement, scruffy fur, cyanotic paws and mouth, and poor mental response. The most obvious manifestations were observed 1–5 days after the induction of pulmonary fibrosis.

Weights of all guinea pigs in both groups increased over time. However, the growth rate of animals in the sham operation group was faster than that of those in the model group. The average weight of the guinea pigs in the model group was significantly lower than that of those in the sham operation group, as measured on the 14th, 21st, and 28th day after the administration of bleomycin (*P* < 0.05) (Table 2, Fig. 1b).

**Table 2:** Comparison of the changes in the body weights in the two groups

Group	Before molding	7th day	14th day	21st day	28th day
Sham	280.15 ± 11.54	319.71 ± 10.92	374.72 ± 16.39	427.02 ± 26.54	463.21 ± 24.10
Model	271.844 ± 6.50	290.61 ± 11.13	322.66 ± 13.02*	345.35 ± 21.50*	365.85 ± 22.53*

### Bleomycin-induced pulmonary fibrosis

Histological staining with H&E and Masson’s trichrome revealed that the alveolar walls were intact and that the alveolar septa showed no thickening in the lung tissue sections of the sham operation group. Furthermore, there was no obvious infiltration of inflammatory cells or collagen deposition in the sham operation group (Fig. 1d, e). The lung tissue sections of the guinea pigs in the model group showed a more severe infiltration of inflammatory cells and fibrosis, thickening of alveolar walls, and distortion of architecture of the pulmonary parenchyma than those of the sham operation group (Fig. 1d, e).

It has been shown that the hydroxyproline content in lung tissues indirectly reflects the collagen content and severity of fibrosis [24]. Correspondingly, the hydroxyproline assay revealed that the concentration of hydroxyproline was significantly higher in the lung tissues of the model group than that of the sham operation group, as measured on the 14th and 28th day after bleomycin administration (Fig. 1f; *P* < 0.05).

Furthermore, unlike those animals of the sham operation group, analysis of Ashcroft fibrotic scores showed that lung tissue sections of the guinea pigs in the model group had significant pulmonary fibrosis on the 14th and 28th days after bleomycin administration (Fig. 1g; *P* < 0.05).

### Cough sensitivity

The cough sensitivity test, which involved the induction of using capsaicin, revealed that the number of coughs was significantly higher in the model group than in the sham operation group on the 7th, 14th, 21st, and 28th day after bleomycin administration (Fig. 1c; *P* < 0. 05).

### Expression of TRPA1 and TRPV1 (mRNA and proteins)

**Fig. 2:**
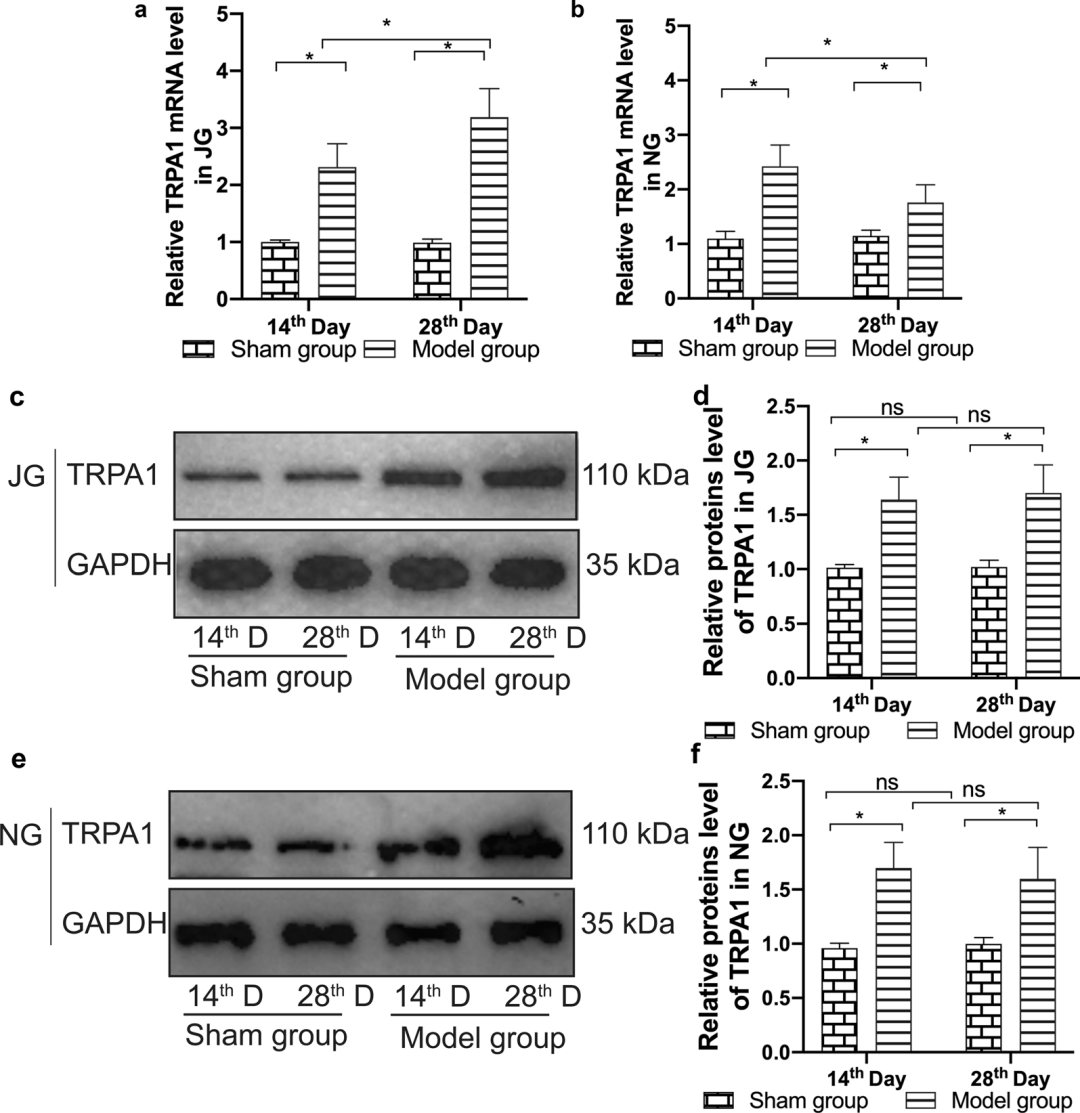
mRNA and protein expression of TRPA1 in the jugular ganglion and nodal ganglion. Expression of mRNA encoding TRPA1 in the jugular ganglion (**a**) and nodal ganglion (**b**) was measured using RT-qPCR, while expression of TRPA1 protein in the jugular ganglion (**c**, **d**) and nodal ganglion (**e**–**f**) was measured using western blotting. Quantitative histograms represent the intensity of the strip optical density (n = 6/group/ per time point). The data are expressed as the mean ± SD. *P < 0.05

**Fig. 3:**
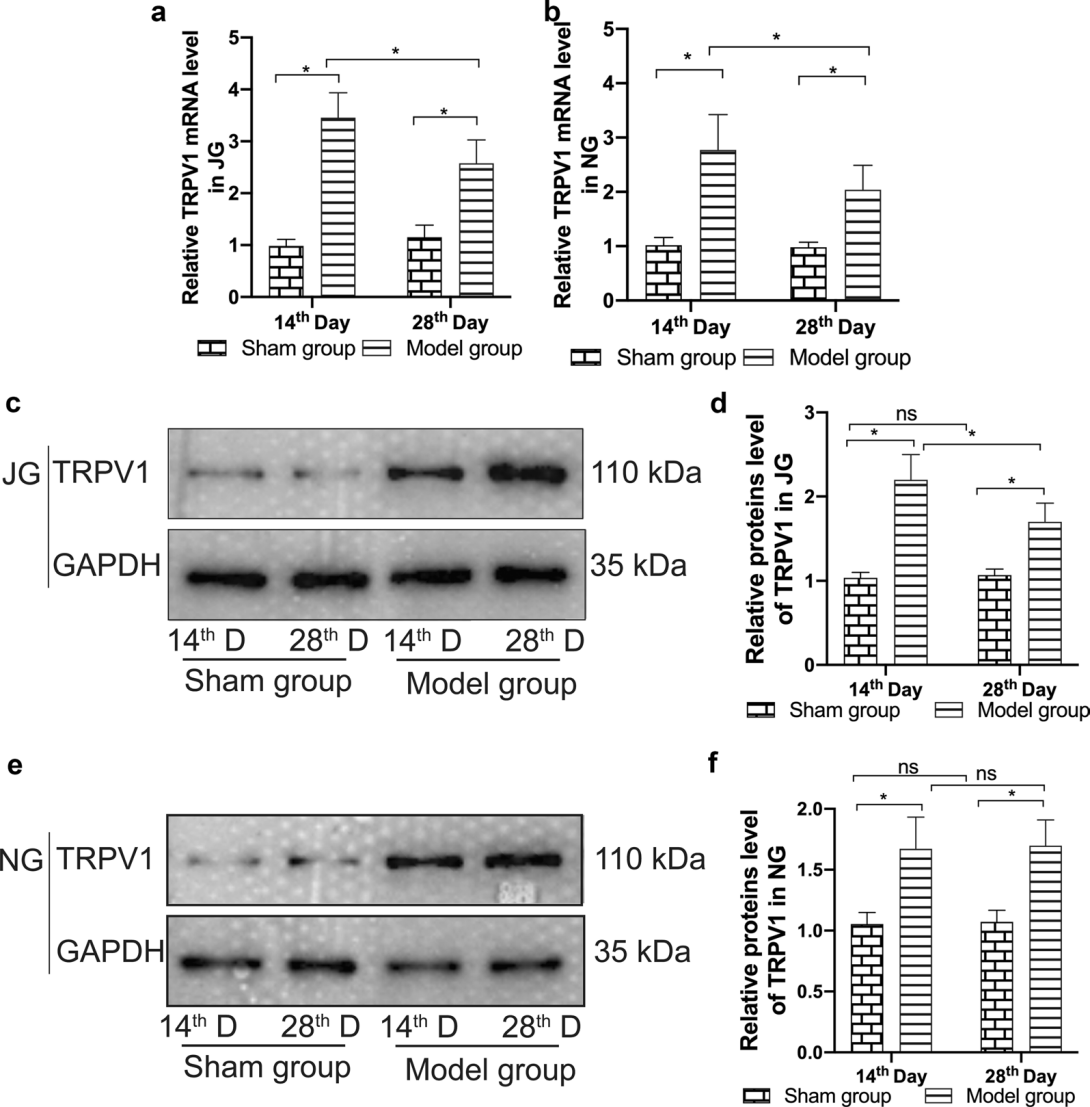
mRNA and protein expression of TRPV1 in the jugular ganglion and nodal ganglion. Expression of mRNA encoding TRPV1 in the jugular ganglion (**a**) and nodal ganglion (**b**) was measured using RT-qPCR, while expression of TRPV1 protein in the jugular ganglion (**c**, **d**) and nodal ganglion (**e**, **f**) was measured using western blotting. Quantitative histograms represent the intensity of the strip optical density (n = 6/group/ per time point). The data are expressed as the mean ± SD. *P < 0.05

RT-qPCR showed that the expression of mRNA encoding TRPA1 and TRPV1 was significantly higher in the jugular ganglion and nodular ganglion tissues of the model group than that of the sham operation group, as measured on the 14th and 28th day after the administration of bleomycin (Figs. 2a, b, 3a, b; *P* < 0.05).

Western blotting revealed that the expression of TRPA1 and TRPV1 proteins in the jugular ganglion and nodular ganglion was significantly higher in the model group than that of the sham operation group on the 14th and 28th day after the administration of bleomycin (Figs. 2c–f and 3c–f; *P* < 0.05).

### Expression of SP, NK1R and CGRP (mRNA and proteins)

**Fig. 4:**
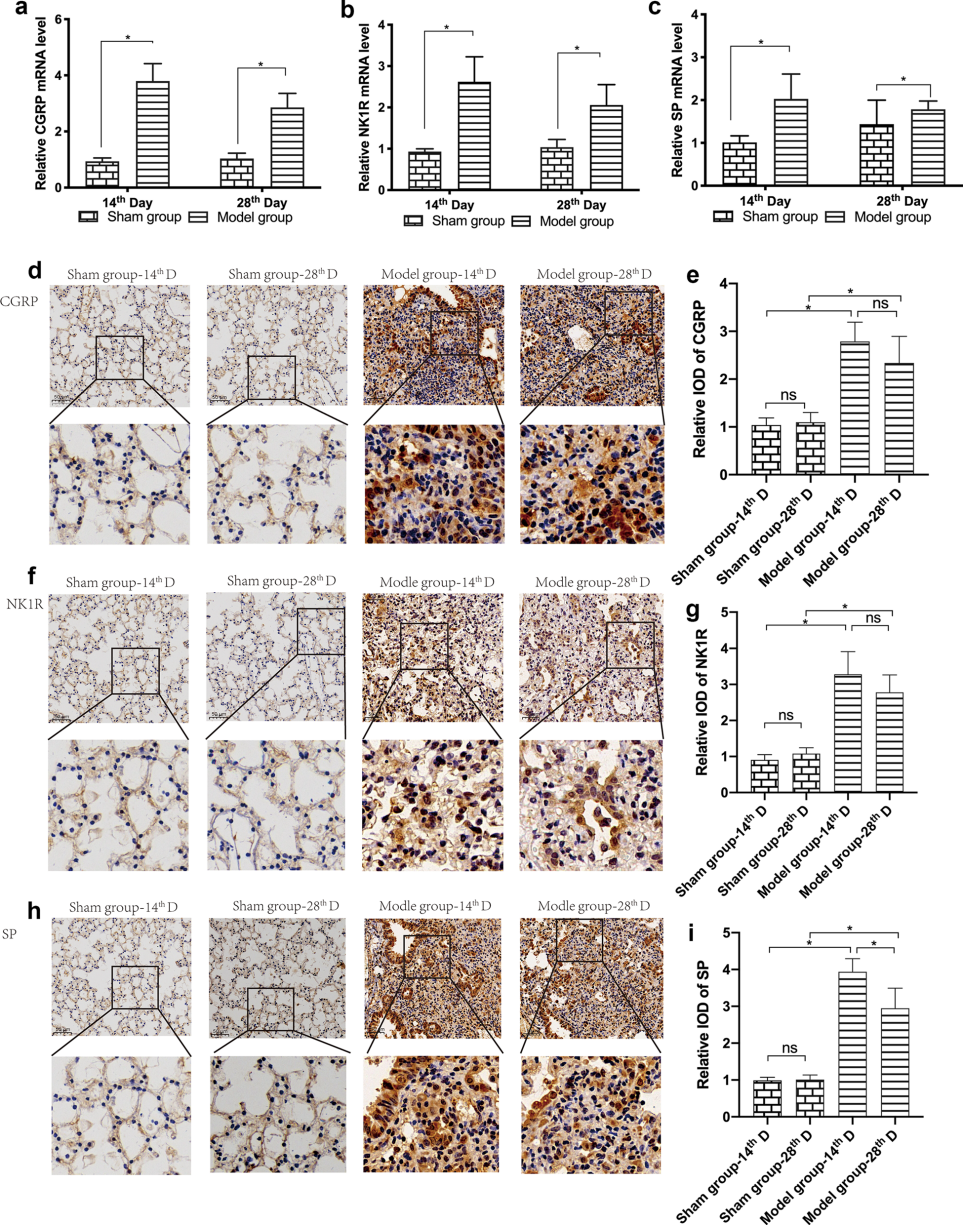
mRNA and protein expression of CGRP, NK1R, and SP in lung tissue. Fold changes in mRNA expression of CGRP (**a**), NK1R (**b**), and SP (**c**) at 14 and 28 days after treatment with bleomycin were detected by RT-qPCR. Immunoreactivity for CGRP (**d**, **e**), NK1R (f-g), and SP (**h**, **i**) in lung sections was measured (original magnification × 20). Quantitative analysis was performed by the integrated optical density (IOD) values using IPP software (n = 6/group). The data are expressed as the mean ± SD. *P < 0.05

Results of RT-qPCR showed that expression of mRNA encoding SP, NK1R, and CGRP was significantly higher in the lung tissues of the model group than those of the sham operation group, as measured on the 14th and 28th day after the administration of bleomycin (Fig. 4a–c; *P* < 0.05).

Immunohistochemical staining revealed that the immunoreactivity for SP, NK1R, and CGRP was also significantly higher in the lung tissue sections of the model group on the 14th and 28th day after the administration of bleomycin than those of in the sham operation group (Fig. 4d–i; *P* < 0.05).

### Concentrations of NKA, NKB, NKF and BDNF

**Fig. 5:**
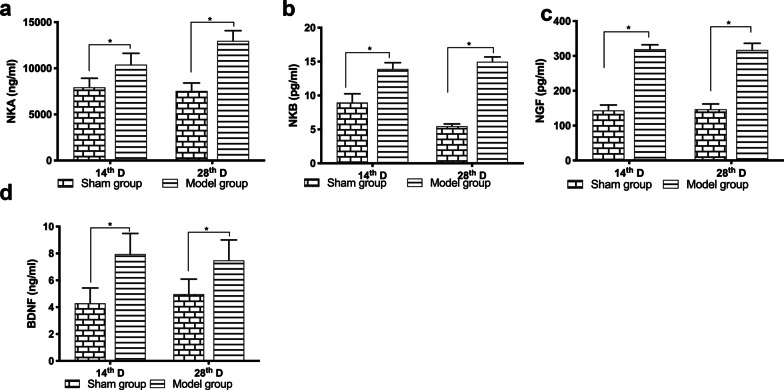
Changes in concentrations of neurotrophic factors in lung tissue homogenates. Changes in the concentrations of NKA (**a**), NKB (**b**), NGF (**c**), and BDNF (**d**) in lung tissue homogenate samples, as measured by ELISA (n = 7–8 animals/group/ per time point). The data are expressed as the mean ± SD. *P < 0.05

Results of ELISA revealed that the concentrations of NKA, NKB, NGF, and BDNF were significantly greater in the lung samples of the model group on the 14th and 28th day after the administration of bleomycin than those of the sham operation group (Fig. 5a–d; *P* < 0.05).

### Correlation analysis

**Fig. 6:**
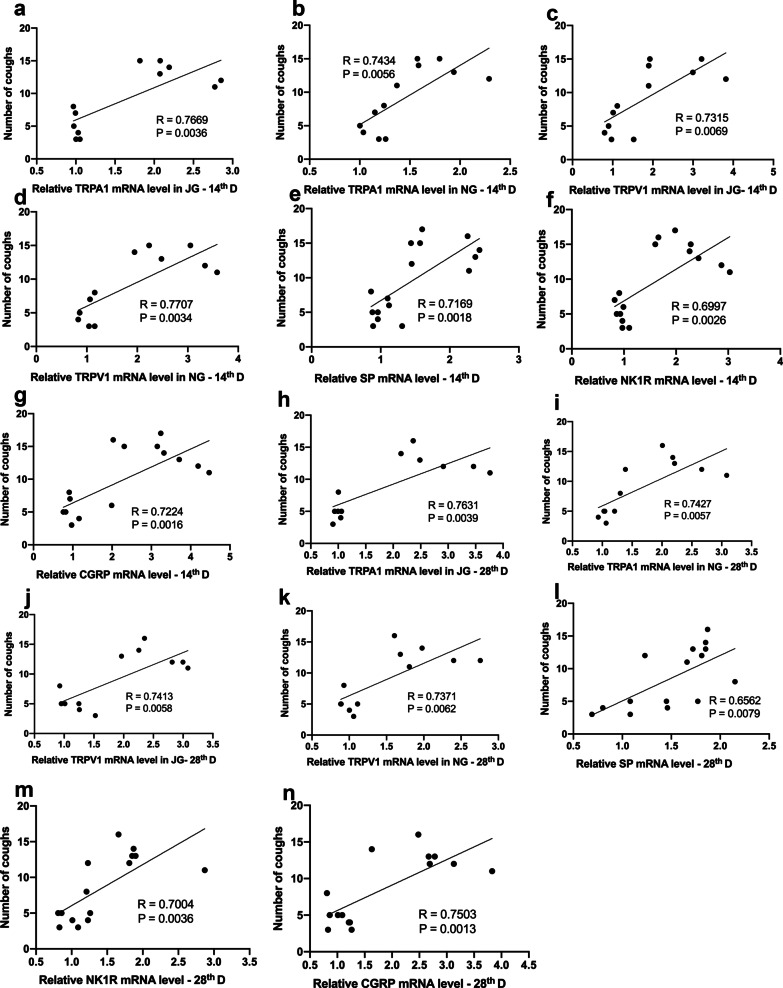
Correlation analysis. The number of coughs with the relative expression of mRNA encoding TRPA1 and TRPV1 in the jugular ganglion (**a**, **c**, **h**, **j**) and nodular ganglion (**b**, **d**, **i**, **k**) of experimental animals on days 14 and 28. Correlation of relative expression of SP (**e**, **i**), NK1R (**f**, **m**), and CGRP (**g**, **n**) in lung tissue on day 14 and day 28 with the number of coughs. The data were obtained by using a Pearson correlation coefficient

To further explore whether the cough sensitivity is associated with the relevant indexes measured, we analyzed correlations between the expression of TRP channels and neurogenic factors SP, NK1R, and CGRP and cough numbers of the experimental animals at days 14 and 28. Results showed that there was a positive correlation between the number of coughs and the expression of mRNA encoding TRPV1 and TRPA1 in the jugular ganglion (JG) and nodular ganglion (NG) on day 14 and day 28 (Fig. 6, day 14: a-d, a, TRPA1 (JG): R^2^ = 0.7669, *p* = 0.0036; b, TRPA1 (NG): R^2^ = 0.7434, *p* = 0.0056; c,TRPV1 (JG): R^2^ = 0.7315, *p* = 0.0069; d, TRPV1 (NG): R^2^ = 0.7707, *p* = 0.0034; day 28: h–k, h, TRPA1 (JG): R^2^ = 0.7631, *p* = 0.0039; i,TRPA1 (NG): R^2^ = 0.7427, *p* = 0.0057; j, TRPV1 (JG): R^2^ = 0.7413, *p* = 0.0058; k, TRPV1 (NG): R^2^ = 0.7371, *p* = 0.0062). In addition, the number of coughs also positively correlated with expression of mRNA encoding SP, NK1R, and CGRP on day 14 and day 28 (Fig. 6, day 14:e–g, e, SP: R^2^ = 0.7169, *p* = 0.0018; f, NK1R: R^2^ = 0.6997, *p* = 0.0026; g, CGRP: R^2^ = 0.7224, *p* = 0.0016; day 28: l-n, l, SP: R^2^ = 0.6562, *p* = 0.0079; m, NK1R: R^2^ = 0.7004, *p* = 0.0036; n, CGRP: R^2^ = 0.7503, *p* = 0.0013). Furthermore, expression of mRNA encoding TRP channels also positively correlated to that of SP, NK1R, and CGRP (The correlation coefficients and P values are shown in Additional file 1: Table 1 and Table 2).

## Discussion

Cough is a common clinical symptom of IPF [25] that seriously affects the quality of life of affected patients; however, its management is limited because of the lack of effective treatment [26]. Using a guinea pig model of bleomycin-induced pulmonary fibrosis with increased cough sensitivity, we explored the changes in the expression of “cough receptors” and mediators involved in airway neurogenic inflammation. Data showed that on the 14^th^ and 28^th^ day after the administration of bleomycin, lung fibrosis, cough sensitivity, and expression of neurogenic inflammatory mediators SP, NK1R, CGRP, NKA, NKB, NKF, and BDNF in the lungs, as well as expression of TRPV1 and TRPA1 in the cough pathway, were significantly elevated. These suggest that there is a relationship between increased cough sensitivity and elevated expression of TRP channels and neurogenic inflammation in pulmonary fibrosis, at least in this guinea pig models. To support this, it worth noting that expression of mRNA encoding TRP channels and neurogenic factors SP, NK1R, and CGRP positively correlated to the number of coughs in the experimental animals.

Current research suggests that increased cough sensitivity is involved in the pathogenesis of IPF-related cough. Doherty et al. [27] have reported that patients with IPF have increased cough sensitivity to inhaled SP and capsaicin, in which the cough-causing capsaicin concentration in the IPF group was much lower than that of the control group. Our data not only showed that the number of coughs in guinea pigs increased significantly after administration of bleomycin, but also that at the same concentration of atomized capsaicin, guinea pigs in the model group coughed sooner and more frequently than those of the sham group, suggesting that guinea pigs with bleomycin-induced pulmonary fibrosis have increased cough sensitivity.

As it has been known, TRPA1 and TRPV1 are closely related to the occurrence of high cough sensitivity. Clarke et al*.* [28] have shown that the expression of TRPV1 on the nerve fibers of the respiratory tract in patients with chronic cough is four times higher than that of healthy people. Our previous study also showed that expression of TRPV1/TRPA1 was upregulated in a guinea pig model of bleomycin-induced pulmonary fibrosis with chronic cough [15]. Every physiological cough requires a complete neural reflex arc, which starts with the activation of afferent fibers of the vagus nerve in the airway [17, 29]. Therefore, any changes or abnormalities in the components of the reflex arc can cause abnormal cough reflexes and change cough sensitivity [30]. In fact, the cough receptors (i.e., sensory nerve endings) distributed in the airway include C fiber endings, fast-adapting receptors, and slow-adapting receptors. Their cell bodies are mainly derived from the jugular ganglion, nodose ganglion, and thoracic dorsal root ganglion (DRG). Increased expression of TRPA1\TRPV1 in the jugular ganglion and nodose ganglia of the guinea pig model of bleomycin-induced pulmonary fibrosis suggests that such up-regulated expression of TRPA1\TRPV1 on the cough afferent pathway might be associated with the increased cough sensitivity in animal models; however, a previous study [31] showed that there is unchanged mRNA expression of TRPV1 and TRPA1 in the lung. This inconsistency may be explained by differences in the efficiencies of the primers and in modeling methods. In addition, in this study, TRPV1 played an important role in the etiology of IPF-related cough. However, Belvisi et al. [32] found that TRPV1 antagonists had no effect on the objective cough frequency of patients with refractory chronic cough. This suggests that chronic cough has various types and complex mechanisms. For different types of cough, the role of TRPV1 in the pathogenesis may be different. In IPF-related cough, the development of cough treatment drugs for TRPV1 still needs further investigation.

Previous studies have shown that the levels of SP, NGF, BDNF, and Trk receptors increase, either in BALF, sputum, or in the lung tissue of patients with IPF [13], suggesting that neurogenic airway inflammation plays an important role in the pathogenesis of the disease. It is known that these neurotrophic factors can induce the survival and development of sensory neurons, increase capsaicin sensitivity, and enhance cough reflex. Our ELISA data showed that NKA\NKB levels increased significantly in lung tissue homogenates of the model group, suggesting that neurogenic inflammation of the lung might contribute to the pathogenesis of IPF cough, although the precise role remains to be verified. It is possible that the peripheral lung-tissue fibrotic lesions produce neurotrophic factors that, in turn, act on the near central airway to regulate the growth and survival of neurons and affect neuronal plasticity. In addition, NGF and BDNF can also induce the synthesis of tachykinin in bronchopulmonary C-type fibers [33], thereby directly or indirectly enhancing nerve conductivity and sensitivity and mediating airway neurogenic hyperresponsiveness [26]. In this study, changes in neurogenic inflammation indicators were observed in the guinea pig model; however, it has not been confirmed that neurogenic inflammation plays a role in nerve activation in humans.

The interaction between TRP channels and neurogenic inflammation is complex, and a variety of neurotransmitters and inflammatory mediators can sensitize TRPV1 channels, including SP, NGF, NKA, and NKB. In addition, neurons expressing TRPV1 also contain and release neuropeptides, such as SP, CGRP, NKA, and NKB. Administration of BDNF or ovalbumin into the trachea of guinea pigs increases sensitivity of nodose ganglion cells to capsaicin, indicating that the airway nerves are plastic [34, 35]. We have previously also shown that the expression of TRPV1 and TRPA1 increased in the lungs of guinea pigs with bleomycin-induced pulmonary fibrosis [19]. Correspondingly, our data of the present study showed that the cough sensitivity of guinea pigs was increased on the 14^th^ and 28^th^ days after bleomycin treatment. In the meantime, the expression of TRPA1 and TRPV1 in the jugular ganglia and nodose ganglia, and the expression of CGRP, SP, and NK1R in the lungs, was also elevated. Furthermore, expression of TRP channels and these neurogenic factors significantly correlated each other, at both the early and late stage of development of the animal models. Taken together, these findings further support the concept that TRP channel-related proteins and neurogenic inflammation contribute to cough sensitivity during pulmonary fibrosis.

Many factors might be accountable for pulmonary fibrosis-related cough, such as inflammatory mediators, neuropeptides, oxidative stress, reactive oxygen species (ROS), and fibrotic mechanical traction [27, 36]. For example, IPF can cause structural distortion of the lungs and fibrotic scars, which theoretically increase the mechanical pressure of the lungs, thus increasing cough sensitivity. It has been shown that patients with IPF have increased cough sensitivity to mechanical stimulation, while changes in patients with IPF can further cause cough-inhibiting nerve damage and impair the negative feedback regulation mechanism of cough [7]. In addition, TRPA1 is also a molecular target of oxidative stress [37], and activating TRPV1/TRPA1 can release inflammatory mediators [38]. This may explain the increased cough sensitivity under disease conditions. In addition to TRPA1 and TRPV1, other ion channels such as TRPV4 and P2X3 may contribute to cough hypersensitivity [39–41]; however, the role of these ion channels in IPF-COUGH needs further investigations.

At present, a few experimental animal models of pulmonary fibrosis have been developed in mice and rats by intratracheal infusion of bleomycin, radiation exposure, and other methods [42]. However, whether mice and rats can cough like humans remains controversial. When constructing animal models of cough, animals with high similarity to humans in the structure of lung tissue and the innervation of the trachea and bronchus are more suitable. Guinea pigs can cough when they are awake and do not restrict their activities. The sound of a guinea pig’s cough is easy to recognize, and the cough reflex produced by guinea pigs to chemical stimuli such as citric acid and capsaicin in the awake state is similar to that of humans [43, 44]. For the above reasons, we used guinea pigs as experimental animals; however, some limitations exist in the present study. Firstly, because of the lack of animal cough detection systems in our lab, the number of coughs was separately counted at the same time by two trained observers who were blinded to status of the animal conditions. This method is time-consuming, labor-intensive, and possibly less accurate. Currently, animal cough detection systems are available for mice, rats, and guinea pigs, which can hopefully increase the accuracy of cough judgment. Secondly, because of the limited time and samples, we could only analyze expression of neurogenic inflammatory indicators in the lung. Subsequent experiments should be further performed to detect the expression of neurogenic inflammatory indicators in the cough pathway, including the jugular ganglia and nodose ganglia. In addition, it can be further explored whether there is a decrease in neurogenic inflammation-related indicators after the use of TRP channel protein inhibitors. Further, ganglion contains a variety of cells such as neurons, satellite cells (a kind of glial cell), and Schwann cells [45]. Due to experimental conditions, we were unable to isolate neurons; therefore, the expression of TRP channel proteins detected in this study is the expression in the entire ganglion.

## Conclusion

In summary, we used bleomycin to induce pulmonary fibrosis in a guinea pig model with high cough sensitivity. The expression of TRPA1 and TRPV1 in the jugular ganglion and nodose ganglia, as well as the expression of SP, NK1R, and CGRP in the lung were elevated. These suggest that the increased expression of TRPA1 and TRPV1 in the cough reflex pathway and neurogenic inflammation may be associated with IPF-related high cough sensitivity.

## Supplementary Information

**Additional file 1: Supplementary Figure 1**. Instruments used for tracheal drug delivery. **Supplementary Figure 2**. Full-length blots of Figures 2 and 3. **Supplementary Table 1**. Correlation analysis between the relative expression of mRNA encoding TRP channel protein and neurotrophic factors at day 14. **Supplementary Table 2**. Correlation analysis between the relative expression of mRNA encoding TRP channel protein and neurotrophic factors at day 28.

## Data Availability

The datasets used and/or analyzed in the current study are available from the corresponding author upon reasonable request.

## Abbreviations

BALF: Bronchoalveolar lavage fluid
BDNF: Brain-derived neurotrophic factor
BLM: Bleomycin
CGRP: Calcitonin gene-related peptide
DRG: Thoracic dorsal root nerve ganglion
IPF: Idiopathic pulmonary fibrosis
JG: Jugular ganglion
NG: Ganglion
NGF: Nerve growth factor
NKA: Neurokinin A
NKB: Neurokinin B
NK1R: Neurokinin-1 receptor
PBS: Phosphate Buffered Saline
RT-qPCR: Real-time quantitative PCR
SEM: Tandard error of the mean
SP: Substance P
TRPA1: TRP channel subfamily A member 1
TRPM8: Transient receptor potential channel subfamily M member 8
TRPV1: TRP channel subfamily vanilloid member 1

## References

[1] Raghu G, Collard HR, Egan JJ, Martinez FJ, Behr J, Brown KK (2011). An official ATS/ERS/JRS/ALAT statement: idiopathic pulmonary fibrosis: evidence-based guidelines for diagnosis and management. Am J Respir Crit Care Med.

[2] Natsuizaka M, Chiba H, Kuronuma K, Otsuka M, Kudo K, Mori M (2014). Epidemiologic survey of Japanese patients with idiopathic pulmonary fibrosis and investigation of ethnic differences. Am J Respir Crit Care Med.

[3] Strand MJ, Sprunger D, Cosgrove GP, Fernandez-Perez ER, Frankel SK, Huie TJ (2014). Pulmonary function and survival in idiopathic vs secondary usual interstitial pneumonia. Chest.

[4] Ryerson CJ, Abbritti M, Ley B, Elicker BM, Jones KD, Collard HR (2011). Cough predicts prognosis in idiopathic pulmonary fibrosis. Respirology.

[5] Lechtzin N, Hilliard ME, Horton MR (2013). Validation of the Cough Quality-of-Life Questionnaire in patients with idiopathic pulmonary fibrosis. Chest.

[6] Vigeland CL, Horton MR (2016). Cough in idiopathic pulmonary fibrosis: more than just a nuisance. Lancet Respir Med.

[7] Jones RM, Hilldrup S, Hope-Gill BD, Eccles R, Harrison NK (2011). Mechanical induction of cough in idiopathic pulmonary fibrosis. Cough.

[8] Sousa-Valente J, Brain SD (2018). A historical perspective on the role of sensory nerves in neurogenic inflammation. Semin Immunopathol.

[9] Sorkin LS, Eddinger KA, Woller SA, Yaksh TL (2018). Origins of antidromic activity in sensory afferent fibers and neurogenic inflammation. Semin Immunopathol.

[10] Xu X, Chen Q, Qiu Z, Shi C, Ding H, Wang L (2018). Association of cough hypersensitivity with tracheal TRPV1 activation and neurogenic inflammation in a novel guinea pig model of citric acid-induced chronic cough. J Int Med Res.

[11] De Logu F, Patacchini R, Fontana G, Geppetti P (2016). TRP functions in the broncho-pulmonary system. Semin Immunopathol.

[12] Taylor-Clark TE (2016). Role of reactive oxygen species and TRP channels in the cough reflex. Cell Calcium.

[13] Chang AB, Gibson PG, Ardill J (2007). Calcitonin gene-related peptide relates to cough sensitivity in children with chronic cough. Eur Respir J.

[14] Hope-Gill BD, Hilldrup S, Davies C, Newton RP, Harrison NK (2003). A study of the cough reflex in idiopathic pulmonary fibrosis. Am J Respir Crit Care Med.

[15] Guo Y, Ying S, Zhao X, Liu J, Wang Y (2019). Increased expression of lung TRPV1/TRPA1 in a cough model of bleomycin-induced pulmonary fibrosis in Guinea pigs. BMC Pulm Med.

[16] Haji A, Kimura S, Ohi Y (2013). A model of the central regulatory system for cough reflex. Biol Pharm Bull.

[17] Canning BJ (2010). Afferent nerves regulating the cough reflex: mechanisms and mediators of cough in disease. Otolaryngol Clin North Am.

[18] Mazzone SB (2004). Sensory regulation of the cough reflex. Pulm Pharmacol Ther.

[19] Canning BJ, Mazzone SB, Meeker SN, Mori N, Reynolds SM, Undem BJ (2004). Identification of the tracheal and laryngeal afferent neurones mediating cough in anaesthetized guinea-pigs. J Physiol.

[20] Zaccone EJ, Lieu T, Muroi T, Potenzieri C, Undem BE, Gao P (2016). Parainfluenza 3-induced cough hypersensitivity in the Guinea pig airways. PLoS ONE.

[21] Tanaka M, Maruyama K (2003). Cough reflex induced by microinjection of citric acid into the larynx of guinea pigs: new coughing model. J Pharmacol Sci.

[22] Ashcroft T, Simpson JM, Timbrell V (1988). Simple method of estimating severity of pulmonary fibrosis on a numerical scale. J Clin Pathol.

[23] Hübner RH, Gitter W, El Mokhtari NE, Mathiak M, Both M, Bolte H (2008). Standardized quantification of pulmonary fibrosis in histological samples. Biotechniques.

[24] Jenkins RG, Moore BB, Chambers RC, Eickelberg O, Königshoff M, Kolb M (2017). An official American Thoracic Society workshop report: use of animal models for the preclinical assessment of potential therapies for pulmonary fibrosis. Am J Respir Cell Mol Biol.

[25] Key AL, Holt K, Hamilton A, Smith JA, Earis JE (2010). Objective cough frequency in idiopathic pulmonary fibrosis. Cough.

[26] Madison JM, Irwin RS (2005). Chronic cough in adults with interstitial lung disease. Curr Opin Pulm Med.

[27] Doherty MJ, Mister R, Pearson MG, Calverley PM (2000). Capsaicin induced cough in cryptogenic fibrosing alveolitis. Thorax.

[28] Clarke R, Monaghan K, About I, Griffin CS, Sergeant GP, El Karim I (2017). TRPA1 activation in a human sensory neuronal model: relevance to cough hypersensitivity?. Eur Respir J.

[29] Canning BJ, Mori N, Mazzone SB (2006). Vagal afferent nerves regulating the cough reflex. Respir Physiol Neurobiol.

[30] Coleridge JC, Coleridge HM (1984). Afferent vagal C fibre innervation of the lungs and airways and its functional significance. Rev Physiol Biochem Pharmacol.

[31] Fernández-Blanco JA, Aguilera M, Domènech A, Tarrasón G, Prats N, Miralpeix M (2015). Enhanced cough reflex in a model of bleomycin-induced lung fibrosis in guinea pigs. Clin Sci (Lond).

[32] Belvisi MG, Birrell MA, Wortley MA (2017). XEN-D0501, a novel transient receptor potential vanilloid 1 antagonist, does not reduce cough in patients with refractory cough. Am J Respir Crit Care Med.

[33] Lindsay RM, Harmar AJ (1989). Nerve growth factor regulates expression of neuropeptide genes in adult sensory neurons. Nature.

[34] Lieu TM, Myers AC, Meeker S, Undem BJ (2012). TRPV1 induction in airway vagal low-threshold mechanosensory neurons by allergen challenge and neurotrophic factors. Am J Physiol Lung Cell Mol Physiol.

[35] Lieu T, Undem BJ (2011). Neuroplasticity in vagal afferent neurons involved in cough. Pulm Pharmacol Ther.

[36] Schelegle ES, Walby WF, Mansoor JK, Chen AT (2001). Lung vagal afferent activity in rats with bleomycin-induced lung fibrosis. Respir Physiol.

[37] Lin YS, Hsu CC, Bien MY, Hsu HC, Weng HT, Kou YR (1985). Activations of TRPA1 and P2X receptors are important in ROS-mediated stimulation of capsaicin-sensitive lung vagal afferents by cigarette smoke in rats. J Appl Physiol.

[38] McGarvey LP, Butler CA, Stokesberry S, Polley L, McQuaid S, Abdullah H (2014). Increased expression of bronchial epithelial transient receptor potential vanilloid 1 channels in patients with severe asthma. J Allergy Clin Immunol.

[39] Bonvini SJ, Birrell MA, Grace MS (2016). Transient receptor potential cation channel, subfamily V, member 4 and airway sensory afferent activation: role of adenosine triphosphate. J Allergy Clin Immunol.

[40] Kamei J, Takahashi Y, Yoshikawa Y (2005). Involvement of P2X receptor subtypes in ATP-induced enhancement of the cough reflex sensitivity. Eur J Pharmacol.

[41] Abdulqawi R, Dockry R, Holt K (2015). P2X3 receptor antagonist (AF-219) in refractory chronic cough: a randomised, double-blind, placebo-controlled phase 2 study. Lancet.

[42] Kolb P, Upagupta C, Vierhout M, Ayaub E, Bellaye PS, Gauldie J (2020). The importance of interventional timing in the bleomycin model of pulmonary fibrosis. Eur Respir J.

[43] Laude EA, Higgins KS, Morice AH (1993). A comparative study of the effects of citric acid, capsaicin and resiniferatoxin on the cough challenge in guinea-pig and man. Pulm Pharmacol.

[44] Lewis CA, Ambrose C, Banner K, Battram C, Butler K, Giddings J (2007). Animal models of cough: literature review and presentation of a novel cigarette smoke-enhanced cough model in the guinea-pig. Pulm Pharmacol Ther.

[45] Shoji Y, Yamaguchi-Yamada M, Yamamoto Y (2010). Glutamate- and GABA-mediated neuron-satellite cell interaction in nodose ganglia as revealed by intracellular calcium imaging. Histochem Cell Biol.

