# Supplementary material for: Increased expression of transient receptor potential channels and neurogenic factors associates with cough severity in a guinea pig model
Source: BMC Pulm Med. 2021 Jun 2;21:187. doi: 10.1186/s12890-021-01556-w (PMC8173754; doi:10.1186/s12890-021-01556-w)
Supplement: Supplementary file 1 — Additional file 1: Supplementary Figure 1. Instruments used for tracheal drug delivery. Supplementary Figure 2. Full-length blots of Figures 2 and 3. Supplementary Table 1. Correlation analysis between the relative expression of mRNA encoding TRP channel protein and neurotrophic factors at day 14. Supplementary Table 2. Correlation analysis between the relative expression of mRNA encoding TRP channel protein and neurotrophic factors at day 28. [file 12890_2021_1556_MOESM1_ESM.docx]

**The title of the manuscript:** **Increased expression of transient receptor potential channels and neurogenic factors associates with cough severity in a guinea pig model**

**The author list:** **Mengyue Guan^a^, Sun Ying^b^, Yuguang Wang^a,*^**

**supplementary information**

**Supplementary Figure 1. Instruments used for tracheal drug delivery**

**
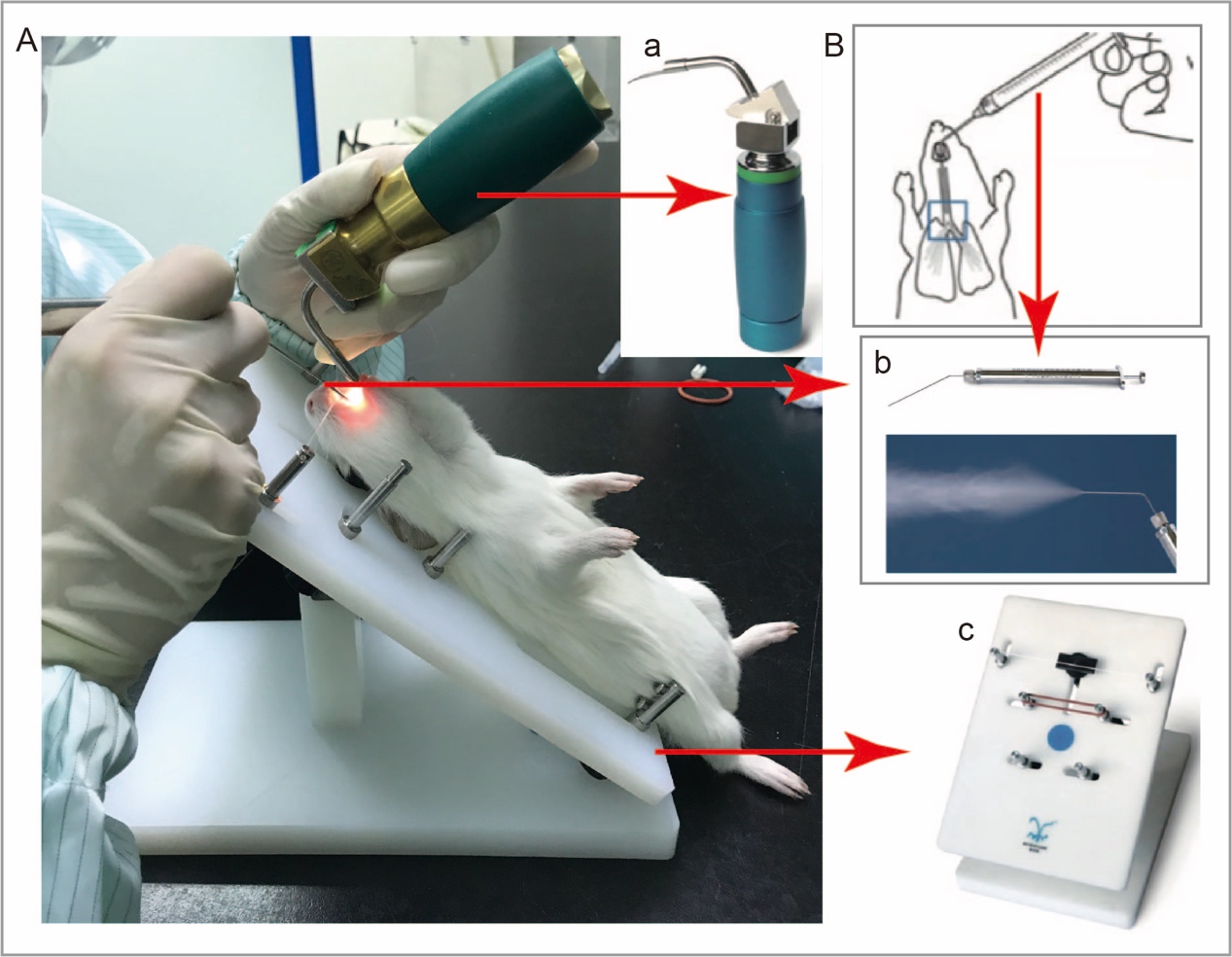
**

For the intratracheal administration of bleomycin (A), the guinea pigs were placed on a fixed table (c), and bleomycin was administered using a small animal laryngoscope (a) and an endotracheal nebulizer (b). The endotracheal nebulizer converted the bleomycin solution into an aerosol form (b) and directly delivered the drug into the lungs (B).

**Supplementary Figure 2. Full-length blots of Figures 2 and 3**

**
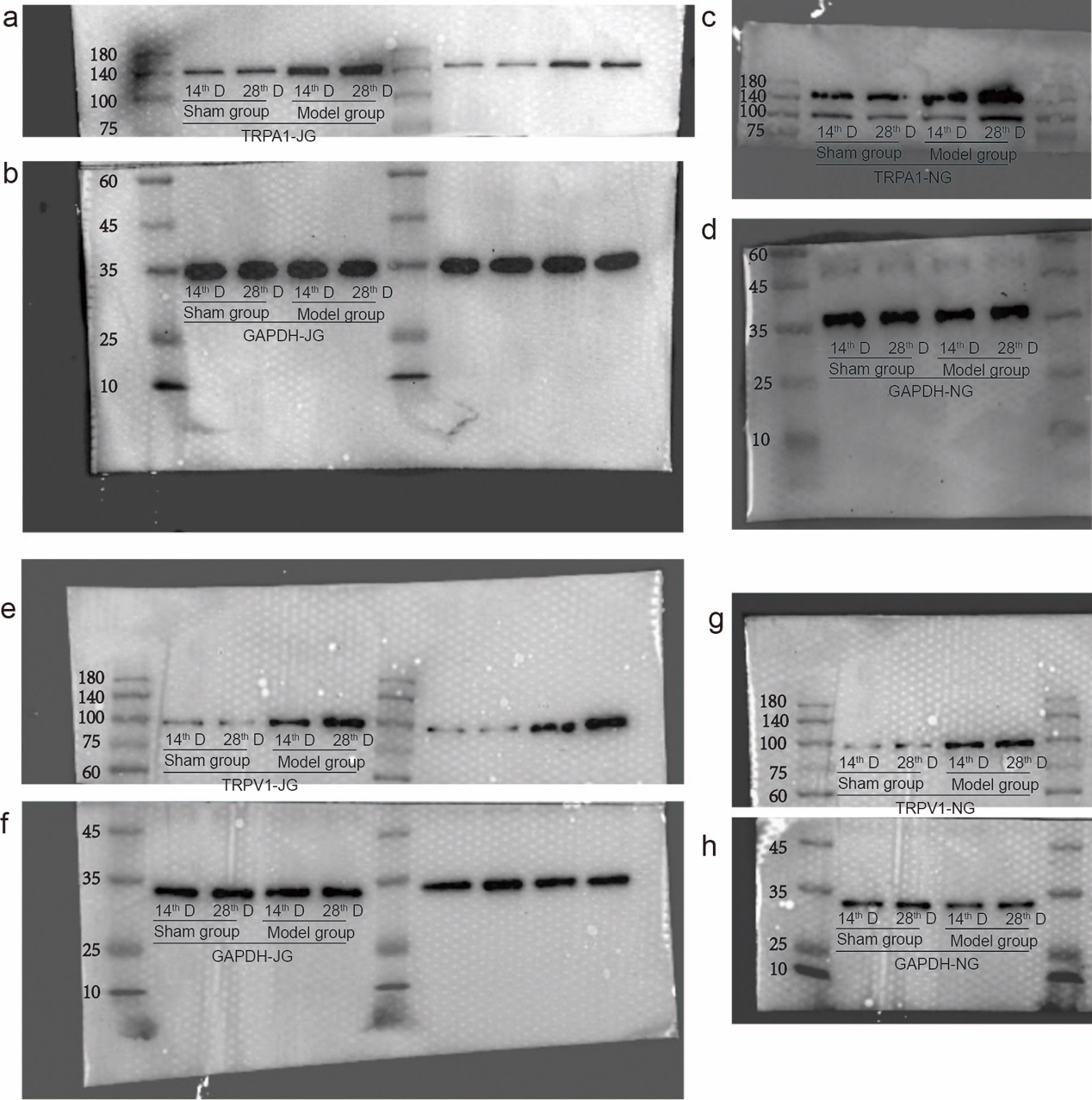
**

Full-length blots of TRPA1 and GAPDH in the in the (a and b) jugular ganglion of Figure 2c and (c and d) nodal ganglion of Figure 2e. Full -length blots of TRPV1 and GAPDH in the (e and f) jugular ganglion of Figure 3c and (g and h) nodal ganglion of Figure 3e.

**Supplementary Table 1. Correlation analysis between the relative expression of mRNA encoding TRP channel protein and neurotrophic factors at day 14**

| 14 Day | TRPA1 JG | TRPA1 NG | TRPV1 JG | TRPV1 NG | SP | NK1R |
| --- | --- | --- | --- | --- | --- | --- |
| TRPA1 NG | R= 0.7837  P= 0.0026 |  |  |  |  |  |
| TRPV1 JG | R= 0.7718  P= 0.0033 | R=0.9633  P< 0.0001 |  |  |  |  |
| TRPV1 NG | R= 0.9561  P= 0.0001 | R=0.7465  P=0.0053 | R=0.7439  P=0.0055 |  |  |  |
| SP | R= 0.7600  P= 0.0041 | R=0.5491  P=0.0645 | R=0.5155  P=0.0863 | R=0.6657  P=0.0181 |  |  |
| NK1R | R= 0.9598  P< 0.0001 | R=0.7935  P=0.0021 | R=0.8170  P=0.0012 | R=0.8962  P< 0.0001 | R=0.8159  P=0.0012 |  |
| CGRP | R= 0.551  P= 0.100 | R=0.7969  P=0.0019 | R=0.8149  P=0.0012 | R=0.9032  P< 0.0001 | R=0.8339  P=0.0007 | R=0.9947  P< 0.0001 |

TRPA1 JG: the relative expression of mRNA encoding TRPA1 in jugular ganglion at day 14; TRPA1 NG: the relative expression of mRNA encoding TRPA1 in nodular ganglion at day 14; TRPV1 JG: the relative expression of mRNA encoding TRPV1 in jugular ganglion at day 14; TRPV1 NG: the relative expression of mRNA encoding TRPV1 in nodular ganglion at day 14; SP: the relative expression of mRNA encoding SP in lung tissue at day 14; NK1R: the relative expression of mRNA encoding NK1R in lung tissue at day 14; CGRP: the relative expression of mRNA encoding CGRP in lung tissue at day 14.

**Supplementary Table 2. Correlation analysis between the relative expression of mRNA encoding TRP channel protein and neurotrophic factors at day 28**

| 28 Day | TRPA1 JG | TRPA1 NG | TRPV1 JG | TRPV1 NG | SP | NK1R |
| --- | --- | --- | --- | --- | --- | --- |
| TRPA1 NG | R= 0.8957  P< 0.0001 |  |  |  |  |  |
| TRPV1 JG | R= 0.9483  P< 0.0001 | R=0.7846  P=0.0025 |  |  |  |  |
| TRPV1 NG | R= 0.8778  P= 0.0002 | R=0.8405  P=0.0006 | R=0.7892  P=0.0023 |  |  |  |
| SP | R= 0.3094  P= 0.3278 | R=0.5177  P=0.0847 | R=0.2018  P=0.5294 | R=0.2753  P=0.3865 |  |  |
| NK1R | R= 0.8164  P=0.0012 | R=0.8007  P=0.0018 | R=0.7194  P=0.0084 | R=0.5319  P=0.0751 | R=0.5176  P=0.0400 |  |
| CGRP | R= 0.9531  P< 0.0001 | R=0.7969  P=0.0019 | R=0.9297  P< 0.0001 | R=7661  P=0.0037 | R=0.9626  P< 0.0001 | R=0.8215  P< 0.0002 |

TRPA1 JG: the relative expression of mRNA encoding TRPA1 in jugular ganglion at day 28; TRPA1 NG: the relative expression of mRNA encoding TRPA1 in nodular ganglion at day 28; TRPV1 JG: the relative expression of mRNA encoding TRPV1 in jugular ganglion at day 28; TRPV1 NG: the relative expression of mRNA encoding TRPV1 in nodular ganglion at day 28; SP: the relative expression of mRNA encoding SP in lung tissue at day 28; NK1R: the relative expression of mRNA encoding NK1R in lung tissue at day 28; CGRP: the relative expression of mRNA encoding CGRP in lung tissue at day 28.
